# Melatonin and Docosahexaenoic Acid Decrease Proliferation of PNT1A Prostate Benign Cells via Modulation of Mitochondrial Bioenergetics and ROS Production

**DOI:** 10.1155/2019/5080798

**Published:** 2019-01-09

**Authors:** Guilherme H. Tamarindo, Daniele L. Ribeiro, Marina G. Gobbo, Luiz H. A. Guerra, Paula Rahal, Sebastião R. Taboga, Fernanda R. Gadelha, Rejane M. Góes

**Affiliations:** ^1^Institute of Biology, State University of Campinas, Campinas, SP, Brazil; ^2^Department of Histology, Institute of Biomedical Sciences, Federal University of Uberlândia, Uberlândia, MG, Brazil; ^3^Department of Biology, Institute of Biosciences, Humanities and Exact Sciences, São Paulo State University, São José do Rio Preto, SP, Brazil; ^4^Department of Biochemistry and Tissue Biology, Institute of Biology, State University of Campinas, Campinas, SP, Brazil

## Abstract

Prostate cancer development has been associated with changes in mitochondrial activity and reactive oxygen species (ROS) production. Melatonin (MLT) and docosahexaenoic acid (DHA) have properties to modulate both, but their protective role, mainly at early stages of prostate cancer, remains unclear. In this study, the effects of MLT and DHA, combined or not, on PNT1A cells with regard to mitochondria bioenergetics, ROS production, and proliferation-related pathways were examined. Based on dose response and lipid accumulation assays, DHA at 100 *μ*M and MLT at 1 *μ*M for 48 h were chosen. DHA doubled and MLT reduced (40%) superoxide anion production, but coincubation (DM) did not normalize to control. Hydrogen peroxide production decreased after MLT incubation only (*p* < 0.01). These alterations affected the area and perimeter of mitochondria, since DHA increased whereas MLT decreased, but such hormone has no effect on coincubation. DHA isolated did not change the oxidative phosphorylation rate (OXPHOS), but decreased (*p* < 0.001) the mitochondrial bioenergetic reserve capacity (MBRC) which is closely related to cell responsiveness to stress conditions. MLT, regardless of DHA, ameliorated OXPHOS and recovered MBRC after coincubation. All incubations decreased AKT phosphorylation; however, only MLT alone inhibited p-mTOR. MLT increased p-ERK1/2 and, when combined to DHA, increased GSTP1 expression (*p* < 0.01). DHA did not change the testosterone levels in the medium, whereas MLT alone or coincubated decreased by about 20%; however, any incubation affected AR expression. Moreover, incubation with luzindole revealed that MLT effects were MTR1/2-independent. In conclusion, DHA increased ROS production and impaired mitochondrial function which was probably related to AKT inactivation; MLT improved OXPHOS and decreased ROS which was related to AKT/mTOR dephosphorylation, and when coincubated, the antiproliferative action was related to mitochondrial bioenergetic modulation associated to AKT and ERK1/2 regulation. Together, these findings point to the potential application of DHA and MLT towards the prevention of proliferative prostate diseases.

## 1. Introduction

Despite its multifactorial etiology, progression and aggressiveness of prostate cancer (PCa) have been related to oxidative stress [1, 2] and the increased production of reactive oxygen species (ROS) is closely associated to alterations in the mitochondria [3]. Such organelles play a crucial role in all stages of malign transformation [3] and have been associated to PCa due to reduction in apoptotic potential [4], pathogenic mutations in genes encoding the electron transport chain (ETC) respiratory complexes, and loss of mitochondrial DNA and integrity [5]. Therefore, modulation of mitochondria physiology may be a good therapeutic target, either in the prevention of tumor development or in the induction of cancer cell death.

Melatonin (MLT) is a pleiotropic hormone with antioxidant properties that regulate mitochondrial activity [6–10] and has been investigated as a PCa suppressor [11]. Patients with PCa exhibit low MLT serum levels when compared to healthy individuals, with a notable decrease when benign prostatic hyperplasia (BPH) progresses to adenocarcinoma [11, 12]. Most cases of PCa (75%) are diagnosed in men over 65 years [11], coincidental to the period when MLT synthesis is reduced [13] and mitochondrial dysfunction increases due to ROS production [14, 15]. Regarding this evidence, MLT supplementation in patients within risk age of PCa (30–40 years old) may be an interesting chemoprevention strategy [16]. Apart from its own anticancer properties, MLT has also been investigated in combination with other compounds, due to its ability to sensitize cells and potentialize the antiproliferative effect of these compounds by inhibition of survival pathways, e.g., AKT [17]. In this context, polyunsaturated fatty acids omega-3 (PUFA *ω*-3), mainly docosahexaenoic acid (DHA), have been reported to increase mitochondrial ROS in tumor cells through AKT/mTOR inhibition [18], leading to cell death. Furthermore, DHA catabolism occurs in the peroxisome and mitochondria [19] and is very susceptible to oxidation caused by reactive species [20] leading to generation of cyto- and genotoxic hydroperoxides and aldehydes [21]. Although evidence suggests that DHA, a fatty acid present in the human diet and usually adopted as nutritional supplementation, can exert antiproliferative effects, it has not yet been investigated in early stages of prostate proliferative disorders, such as benign hyperplasia.

The treatment adopted in most cases of androgen-dependent PCa is androgen ablation, which can lead to selective advantage to androgen-independent cancer cells that eventually proliferate and promote the recurrence of a more aggressive phenotype [22]. Administration of compounds that affect sexual steroid pathways also have been a strategy in prevention and treatment of PCa, but the mechanisms triggered are still not fully elucidated and their safety is uncertain [23]. In this context, one of the avenues to decrease the high mortality rate caused by PCa [24] may be the chemoprevention focused on the suppression of growth or survival of abnormal cells at early stages. Therefore, our aim was to test, *in vitro*, the antiproliferative potential of MLT and DHA, combined or not, in early stages of prostate proliferative disorders. For this purpose, the proliferation of PNT1A cells, an epithelial benign prostate line sensitive to androgen, and the correlations to ROS production, mitochondrial bioenergetics, and proliferative signaling pathways were evaluated.

## 2. Material and Methods

### 2.1. Cell Culture and Treatments

PNT1A cells (#95012614—Health Protection Agency, England, UK) were seeded in RPMI 1640 medium (#R6504—Sigma-Aldrich, St. Louis, Missouri, EUA) enriched with 10% fetal bovine serum (#S0011—Vitrocell, Campinas, São Paulo, Brazil), 1% of penicillin, streptomycin, and amphotericin B (#15240062—Life Technologies, Paisley, UK) and incubated in a wet incubator with 5% of CO_2_ at 37°C. For cell maintenance, the medium was replaced every 2–3 days and subculture was done when 70–85% confluence was reached. For the experiments, the cells were incubated at the desired density and were allowed to attach during 24 hours.

DHA (#D2534—Sigma-Aldrich, St. Louis, Missouri, EUA) concentrations (10 *μ*M, 20 *μ*M, 50 *μ*M, and 100 *μ*M) were freshly prepared in RPMI 1640 culture medium from a stock solution of 20 mM DHA in anhydrous ethanol (vehicle), and the effects on cell proliferation at 24 h, 48 h, and 72 h were tested. These concentrations were chosen according to previous experiments with normal epithelial prostatic cells RWPE-1 [25]. DHA concentration and time of incubation that first exerted antiproliferative effects, combined to higher lipid accumulation, were chosen to perform all forward assays.

Because there are several studies reporting major pathways triggered by MLT (#M5250—Sigma-Aldrich, St. Louis, Missouri, EUA) in different concentrations [26, 27], we first evaluated physiologic (pM–nM), supraphysiologic (nM–*μ*M), and pharmacologic (*μ*M–mM) ranges on cell proliferation. Then, the effects of 1 pM, 1 nM, 1 *μ*M, and 1 mM of MLT were tested alone or in coincubation with DHA. These MLT concentrations in the medium were freshly prepared from a stock solution at 100 mM in anhydrous ethanol (vehicle), and the first that exerted antiproliferative effects, alone and combined to DHA, were chosen for forward assays. For coincubation assays, DHA and MLT at the desired concentration were added to the medium at the same time. Proliferation assay with luzindole (#15998—Cayman Chemical, MI, USA), a nonselective antagonist of MTR1 and MTR2 membrane receptors, 100-fold higher than the MLT work concentration, was performed in control (vehicle incubation only), melatonin (MLT), DHA, and coincubation (DM) to determine the pathways of hormone action.

To ensure that the mechanisms observed were not due to vehicle, all control assays were incubated with anhydrous ethanol at the same volume of DHA, MLT, or DM added to the cells. Also, the concentration of vehicle in the medium never exceeded 0.5% which did not exert alteration on cell proliferation (data not shown).

### 2.2. Cell Proliferation Assay

The effects of treatments were evaluated after incubation of 1 × 10^4^ cells/well with commercial colorimetric kit CellTiter 96® AQueous One Solution Cell Proliferation Assay (#G3580—Promega Corporation, Madison, WI, USA), according to the manufacturer's instructions. Absorbance was determined at 490 nm in an Epoch microplate reader (BioTek Instruments Inc., Winooski, VT, EUA). Three independent experiments in triplicate were performed for statistical analysis.

### 2.3. Lipid Accumulation

Qualitative evaluation of lipid uptake was assessed by light microscopic analysis after incubation with Oil Red O (#O0625—Sigma-Aldrich, St. Louis, Missouri, EUA). Cells were seeded at 1 × 10^4^ cells/well followed by incubation with DHA (10 *μ*M, 20 *μ*M, 50 *μ*M, and 100 *μ*M) or vehicle (control). After incubation, the medium was removed, and the cells were washed with PBS and fixed with paraformaldehyde 4% for 10 minutes. Then, cells were immersed in isopropanol 60% for 5 minutes at room temperature (RT) followed by incubation for 60 minutes with fresh Oil Red O prepared at 0.03% in isopropanol. After two independent experiments performed in duplicate, the most representative profile of lipid accumulation was considered for each DHA concentration.

Quantitative evaluation of intracellular lipids was assessed after incubation (5 × 10^4^ cells/well) with 5 *μ*M of BODIPY™ Lipid Probes 493/503 (#D3922—Molecular Probes®, Invitrogen) prepared in serum-free medium for 5 minutes at RT, according to the manufacturer's instructions. Immediately, images were captured with an inverted fluorescence microscope (Axio Vert.A1 Carl Zeiss AG, DE) with the same time of exposure (280 ms). Quantification of total fluorescence intensity was assessed with ImageJ software, and at least four hundred cells per treatment from three consecutive passages were considered.

### 2.4. ROS Determination

Hydrogen peroxide (H_2_O_2_) production was determined after incubation of 10^6^ cells/mL in PBS/Mg^+2^, 5 mM of succinate, 1 U/mL of HRP, and 25 *μ*M of Amplex Red (Molecular Probes®, Thermo Fisher Inc.). Fluorescence intensity was assessed at 563 nm excitation and 587 nm emission, using a Cytation 3 Cell Imaging Multi-Mode Reader (BioTek Instruments Inc., Winooski, VT, EUA). Cell H_2_O_2_ production was correlated to fluorescence intensity and calculated as described previously [28]. Three independent experiments in triplicate were considered for statistical analysis.

Superoxide anion production (O_2_^●−^) was assessed after incubation with MitoSOX® Red dye (#M36008—Molecular Probes, Thermo Fisher Inc.), according to the manufacturer's instructions. After treatments, the medium was removed, and the cells (5 × 10^4^/well) were washed twice with KH buffer (NaCl 120 mM, NaHCO_3_ 15 mM, KCl 5 mM, NaH_2_PO_4_ 1.5 mM, and Na_2_HPO_4_ 0.7 mM) and incubated with the dye at 3 *μ*M for 15 minutes at 37°C in an incubator with 5% CO_2_. Then, cells were washed twice with KH buffer to remove the dye that did not enter the mitochondria. A total of fifteen images from wells containing cells of consecutive passages were captured at the same exposure time (1200 ms) with an inverted fluorescence microscope (Axio Vert.A1 Carl Zeiss AG, DE). Total field fluorescence intensity was measured with ImageJ software and normalized with the number of cells per field. At least 250 cells were counted in each treatment.

### 2.5. Morphometric Analysis of Mitochondria

Cells were seeded at a density 5 × 10^4^ cells/well in a CELL^view^ bottom glass (Greiner Bio-One) and, after treatments, incubated with MitoTracker Orange CMTMRos (#M7510 Molecular Probes, Invitrogen) at 50 nM in a serum-free medium at 37°C and 5% CO_2_, according to the manufacturer's instructions. After 15 minutes, media were replaced and live cells were immediately analyzed with an inverted fluorescence microscope (Axio Observer Z1 Carl Zeiss AG, DE). Morphometric evaluation of mitochondria was done with a macro created for ImageJ software by Dagda and colleagues [29]. This tool was previously validated [29] and allows measuring mitochondria stained with the specific fluorescent dye and determining the area and perimeter of the organelle. Measurements were performed in at least fifteen images captured with 40x objective. Fluorescent regions of each image were measured followed by normalization of number of cells per field. At least 100 cells were considered for statistical analysis.

### 2.6. Mitochondrial Membrane Potential Determination

After incubations, cells were centrifuged at 2000 rpm for 5 minutes at 4°C, then the pellets were washed and resuspended in PBS. Respiration buffer (sucrose 125 mM, KCl 65 mM, HEPES 10 mM pH 7.2, MgCl_2_ 1 mM, KH_2_PO_4_ 2 mM, EGTA 0.5 mM, and BSA 0.15%), 10^6^ cells/mL, succinate 5 mM, safranin O 5 *μ*M, and digitonin 20 *μ*M were added to a cuvette, as described by Holden and Sze [30] (concentrations of cells, succinate, safranin O, and digitonin were adapted for increased effectiveness without damaging the integrity of the mitochondria). For analysis, ADP 50 *μ*M, oligomycin 1 *μ*g/mL (OLG), and antimycin A 1 *μ*g/mL (AA) were added to the incubation medium. Values were recorded in a fluorescence spectrophotometer (F-2500 Hitachi) with 495 nm and 589 nm excitation and emission, respectively. The most representative profile of mitochondrial membrane potential of three independent experiments performed in duplicate was plotted and values shown in arbitrary fluorescence units.

### 2.7. Oxygen Consumption Rate

Oxygen consumption rate (OCR) was assessed in a Hansatech Oxygraph (Yellow Springs Corporation) as described by Silva and colleagues [31]. Basal state (S2) was determined after incubation of 10^6^ cells/mL in respiration buffer (sucrose 125 mM, KCl 65 mM, HEPES 10 mM pH 7.2, MgCl_2_ 1 mM, KH_2_PO_4_ 2 mM, EGTA 0.5 mM, and BSA 0.15%) in the presence of digitonin at 20 *μ*M and succinate 5 mM. State 3 (S3) was determined by OCR and assessed after the addition of 50 *μ*M ADP and state 4 (S4) with oligomycin 1 *μ*g/mL. Respiratory control (RC) was calculated as RC = (state 3 OCR/state 4 OCR) and indicates the oxidative phosphorylation rate. ETC maximum velocity (S3U) was determined by OCR and assessed after the addition of mitochondrial uncoupler carbonyl cyanide m-chlorophenyl hydrazone (CCCP) at 1 *μ*M. Mitochondrial bioenergetic reserve capacity was calculated as MBRC = (S3U − S2), as described by Vayalil and Landar [32], considering nonmitochondrial oxygen consumption. At least three experiments in triplicate were considered for statistical analysis.

### 2.8. Testosterone Level in the Medium

After incubations, the medium was removed from the wells and testosterone levels were measured with ELISA assay from an AccuBind Testosterone System Microwells (#3725300—Monobind Inc., Lake Forest, CA, USA) commercial kit, according to the manufacturer's instructions. Concentrations of testosterone (ng/mL) were normalized by protein amount in the cell lysate from the correspondent well. Three independent experiments were performed in triplicate for statistical analysis.

### 2.9. Western Blotting

After incubations, cells (5 × 10^4^/well) were lysed in RIPA buffer in the presence of 10% protease inhibitor cocktail, sodium orthovanadate (1 : 100), and PMSF (1 : 1000). All reagents were obtained from Sigma-Aldrich (St. Louis, Missouri, EUA). Protein quantification was performed in an Epoch microplate reader coupled to Take3 Micro-Volume (BioTek Instruments Inc., Winooski, VT, EUA) at 280 nm. After electrophoresis in SDS-PAGE, proteins (50 or 100 *μ*g/*μ*L) were transferred to the nitrocellulose membrane for 1 hour and 40 minutes in a semidry system. Nonspecific bindings were blocked with BSA 5% in TBST for 1 h (phosphorylated proteins) or nonfat milk at 5% in TBST for 30 minutes (for nonphosphorylated proteins), at RT in a shaker. Primary antibodies anti-phospho-mTOR (rabbit monoclonal, Santa Cruz Biotechnology, 1 : 400), anti-phospho-AKT (rabbit polyclonal, Santa Cruz Biotechnology, 1 : 400), anti-phospho-ERK1/2 (rabbit polyclonal, Cell Signaling, 1 : 1000), anti-GSTP1 (rabbit polyclonal, Cell Signaling, 1 : 1000), and anti-*β*-actin (mouse monoclonal, Santa Cruz Biotechnology, 1 : 1000) were incubated overnight at 4°C. Secondary antibodies were prepared at 10x dilution of the respective primary antibody and incubated at 4°C for 1 h. The ECL system was applied for band visualization and image capture. All incubations were analyzed in triplicate (*n* = 3) in the same gel, and one protein per gel in addition to *β*-actin for loading control was quantified. Relative densitometry was assessed using ImageJ software.

### 2.10. Statistical Analyses

Statistical analyses were performed with GraphPad Prism® software (GraphPad Prism software, v.5.0). First, distribution of samples was analyzed with Kolmogorov-Smirnov and Shapiro-Wilk normality tests. Parametric distributions were submitted to *T* test or one-way ANOVA followed by Tukey test (post hoc); nonparametric distributions to Mann-Whitney or Kruskal-Wallis test followed by Dunn test (post hoc). *p* < 0.05 was considered statistically different.

## 3. Results

### 3.1. Pro- or Antimitogenic Actions of DHA in PNT1A Cells Are Time- and Concentration-Dependent

**Figure 1:**
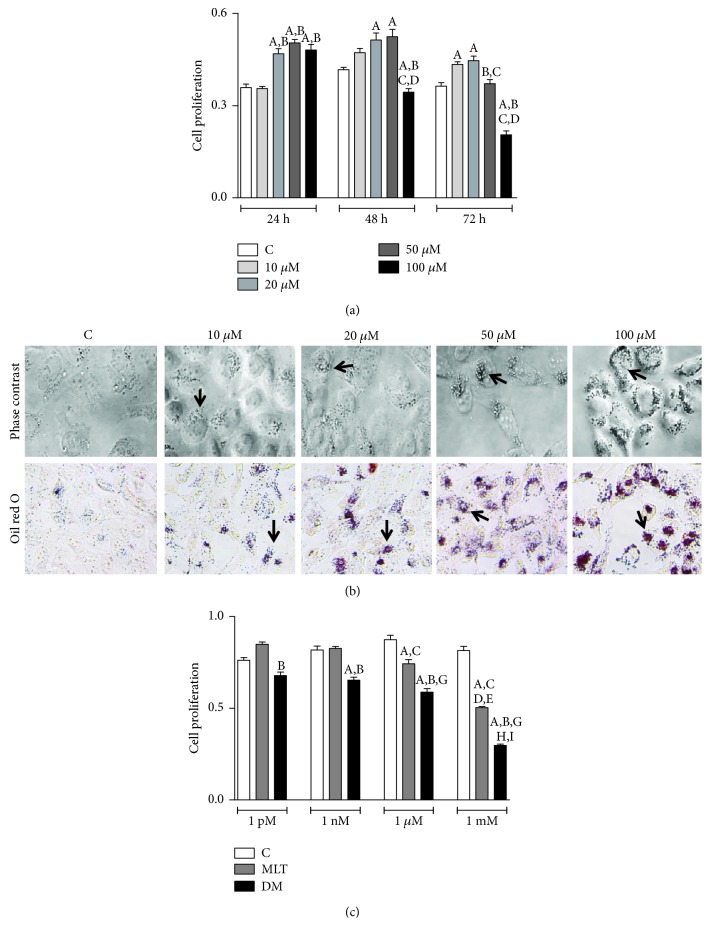
DHA and MLT antiproliferative effect. (a) PNT1A cell proliferation incubated with 10, 20, 50, and 100 *μ*M of DHA after 24, 48, and 72 h. Legend of panel (a): C: control (vehicle incubation). Statistical analysis of panel (a): (A) different from C; (B) different from 10 *μ*M; (C) different from 20 *μ*M; (D) different from 50 *μ*M. (b) Lipid droplets (arrow) stained (red) with Oil Red O (bottom row) after incubation with vehicle (C) or DHA at 10, 20, 50, and 100 *μ*M for 48 h. Images captured with 40x objective. For qualitative analysis, two independent experiments were performed in duplicate, and the most representative lipid accumulation for each DHA concentration is shown. (c) PNT1A cell proliferation incubated with 1 pM, 1 nM, 1 *μ*M, and 1 mM of MLT after 48 h. Legend of panel (c): C: control (vehicle incubation); MLT: melatonin incubation; DM: coincubation with 100 *μ*M of DHA and melatonin. Statistical analysis of panel (c): (A) different from C; (B) different from MLT considering same concentration range; (C) different from MLT-1 pM; (D) different from MLT-1 nM; (E) different from MLT-1 *μ*M; (G) different from DM-1 pM; (H) different from DM-1 nM; (I) different from DM-1 *μ*M; *p* < 0.05 was determined as statistically different. All proliferation assays were performed in triplicate, and three independent events considered for statistical analysis. Values show the mean of absorbance and SEM.

All DHA concentrations tested within 24 h, except 10 *μ*M, increased the proliferation of PNT1A cells. Stimulation of the clonogenic rate at 48 h was only observed with 20 and 50 *μ*M as well as 10 and 20 *μ*M at 72 h (Figure 1(a)). At 100 *μ*M, the highest concentration tested, cell proliferation (Figure 1(a)) reduced only after 48 h (control: 0.41 ± 0.01; DHA 0.34 ± 0.01 abs) and 72 h (control: 0.36 ± 0.01; DHA: 0.20 ± 0.01 abs). DHA also stimulated lipid accumulation in a dose- and time-dependent pattern, as represented in Figure 1(b) for 48 h incubation. Therefore, 100 *μ*M of DHA at 48 h was selected to perform all forward assays.

### 3.2. MLT Decreased PNT1A Cell Proliferation

MLT at physiological concentrations (1 pM and 1 nM) had no effect on cell proliferation (Figure 1(c)) but decreased at 1 *μ*M (control: 0.87 ± 0.02; MLT 0.74 ± 0.02 abs) and 1 mM (control: 0.81 ± 0.02; MLT: 0.50 ± 0.005 abs). At 1 *μ*M, MLT exerted the most antiproliferative effect in comparison to all concentrations analyzed (Figure 1(c)). We chose MLT at the concentration of 1 *μ*M because it was the first to exert an antiproliferative effect. Also, our aim was to evaluate the MLT effects, regardless of membrane receptor activation, which occurs mainly in the pM–nM range.

### 3.3. MLT Improved Antiproliferative Effect When Combined to DHA

MLT coincubated with DHA 100 *μ*M for 48 h roughly decreased cell proliferation (Figure 1(c)) at 1 *μ*M (control: 0.87 ± 0.02; DM: 0.58 ± 0.02 abs) and 1 mM (control: 0.81 ± 0.02; DM: 0.29 ± 0.01 abs). Coincubation with MLT at 1 mM exhibited the most antiproliferative effect, decreasing by 50% the cell proliferation rate compared to DM at 1 *μ*M (Figure 1(c)).

### 3.4. DHA Alone or Coincubated with MLT Increased Lipid Intracellular Amount

**Figure 2:**
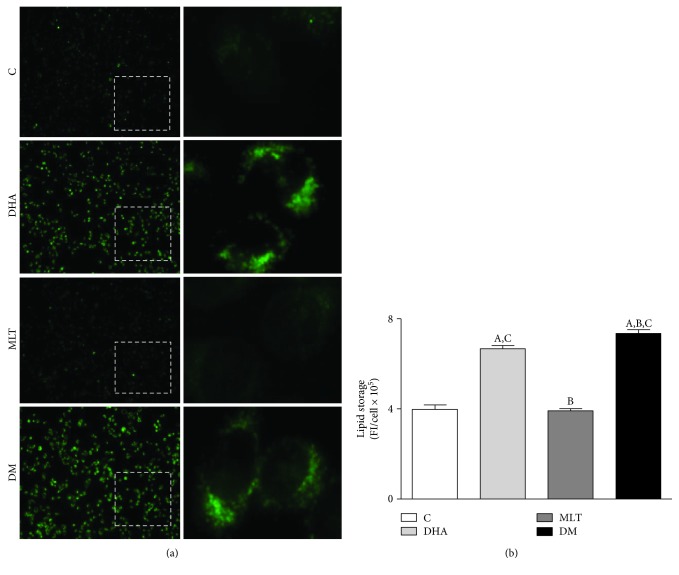
Lipid accumulation. (a) Detection of lipids (green) in PNT1A cells with BODIPY. Cells were incubated with 100 *μ*M of DHA and 1 *μ*M of MLT for 48 h before the dye. Images of left column were captured with 10x objective and right column (insets) with 40x. (b) Lipid storage quantification. C: control (vehicle incubation); DHA: incubation with 100 *μ*M of DHA for 48 h; MLT: incubation with 1 *μ*M of melatonin for 48 h; DM: coincubation with 100 *μ*M of DHA and 1 *μ*M of melatonin for 48 h. Statistical analysis: (A) different from C; (B) different from DHA; (C) different from MLT. *p* < 0.05 was considered statistically different. At least four hundred cells per treatment from three consecutive passages were analyzed. Values show the mean of fluorescent units per cell and SEM.

Incubation with DHA alone (100 *μ*M) increased by 62% lipid accumulation in PNT1A cells after 48 h and, when coincubated with MLT 1 *μ*M, raised to 82% (control: 3.97 ± 0.19, DHA: 6.66 ± 0.14, and DM: 7.34 ± 0.16 FI/cell × 10^5^). MLT alone did not affect intracellular lipid storage (Figure 2).

### 3.5. DHA Increased Superoxide Anion Production and MLT Alleviated ROS Generation

**Figure 3:**
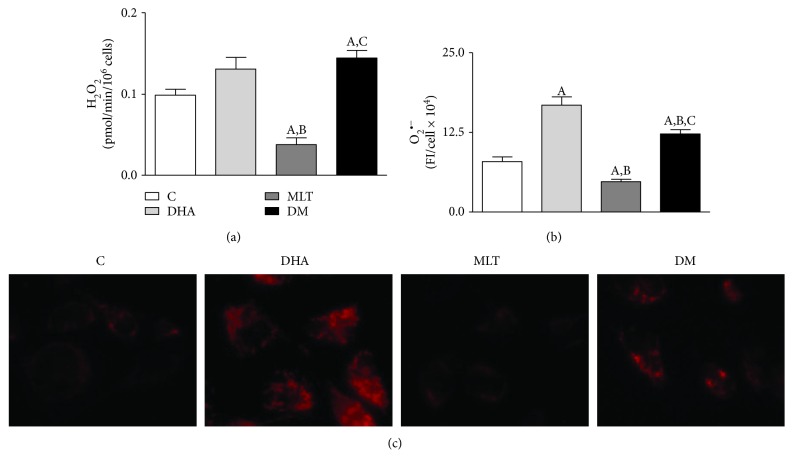
ROS determination. Production of (a) hydrogen peroxide (H_2_O_2_)—values show pmol of H_2_O_2_/min/10^6^ cells and SEM; production of (b) superoxide anion (O_2_^●−^)—values show the fluorescence intensity (FI) per cell × 10^4^. Legend of panels (a) and (b): C: control (vehicle incubation); DHA: incubation with 100 *μ*M of DHA for 48 h; MLT: incubation with 1 *μ*M of melatonin for 48 h; DM: coincubation with 100 *μ*M of DHA and 1 *μ*M of melatonin for 48 h. Statistical analysis: (A) different from C; (B) different from DHA; (C) different from MLT. *p* < 0.05 was considered statistically different. Three independent experiments in triplicate were considered for statistical analysis of H_2_O_2_ production and at least 250 cells for O_2_^●−^. (c) Detection of O_2_^●−^ production after MitoSOX incubation. Images captured with 40x objective with an inverted fluorescence microscope.

DHA did not alter H_2_O_2_ production by PNT1A cells when compared to control (Figure 3(a)). MLT reduced 62% of total H_2_O_2_ production, but when coincubated with DHA, H_2_O_2_ generation increased compared to control, as shown in Figure 3(a) (control: 0.098 ± 0.007, MLT: 0.037 ± 0.008, and DM: 0.144 ± 0.009 pmol/min/10^6^ cells).

DHA doubled whereas MLT alone reduced 40% of superoxide anion production (Figures 3(b) and 3(c)) by PNT1A cells when compared to control. Coincubation attenuated such ROS production compared to DHA alone, but did not normalize to control (control: 7.91 ± 0.73, DHA: 16.78 ± 1.30, MLT: 4.75 ± 0.39, and DM: 12.26 ± 0.65 FI/cell × 10^4^).

### 3.6. MLT Improved Oxidative Phosphorylation and Alleviated Mitochondrial Damage Caused by DHA

**Figure 4:**
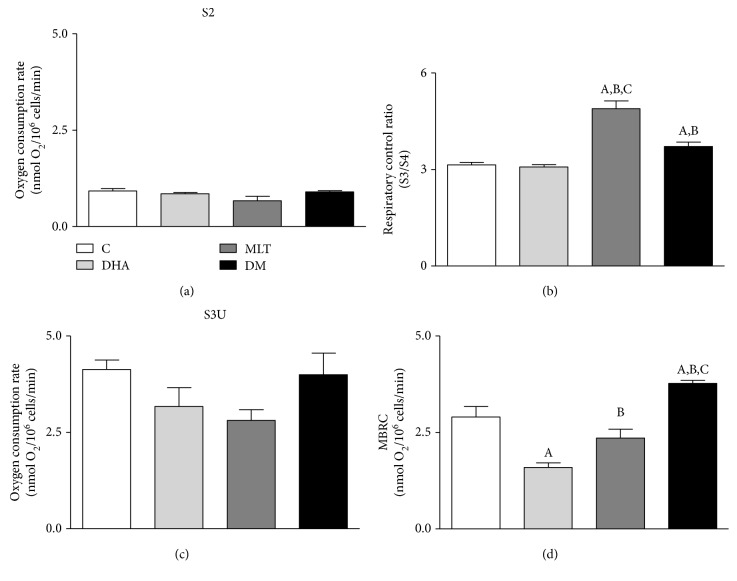
Oxygen consumption rate of PNT1A cells after incubations. (a) Basal state (S2), (b) respiratory control ratio (OCR), (c) maximum velocity of respiratory chain (S3U), and (d) mitochondrial bioenergetic reserve capacity (MBRC). Values show the mean of nmol of O_2_/10^6^ cells/min and SEM. C: control (vehicle incubation); DHA: incubation with 100 *μ*M of DHA for 48 h; MLT: incubation with 1 *μ*M of melatonin for 48 h; DM: coincubation with 100 *μ*M of DHA and 1 *μ*M of melatonin for 48 h. Statistical analysis: (A) different from C; (B) different from DHA; (C) different from MLT. *p* < 0.05 was considered statistically different. Three independent experiments were performed in triplicate for statistical analysis.

**Figure 5:**
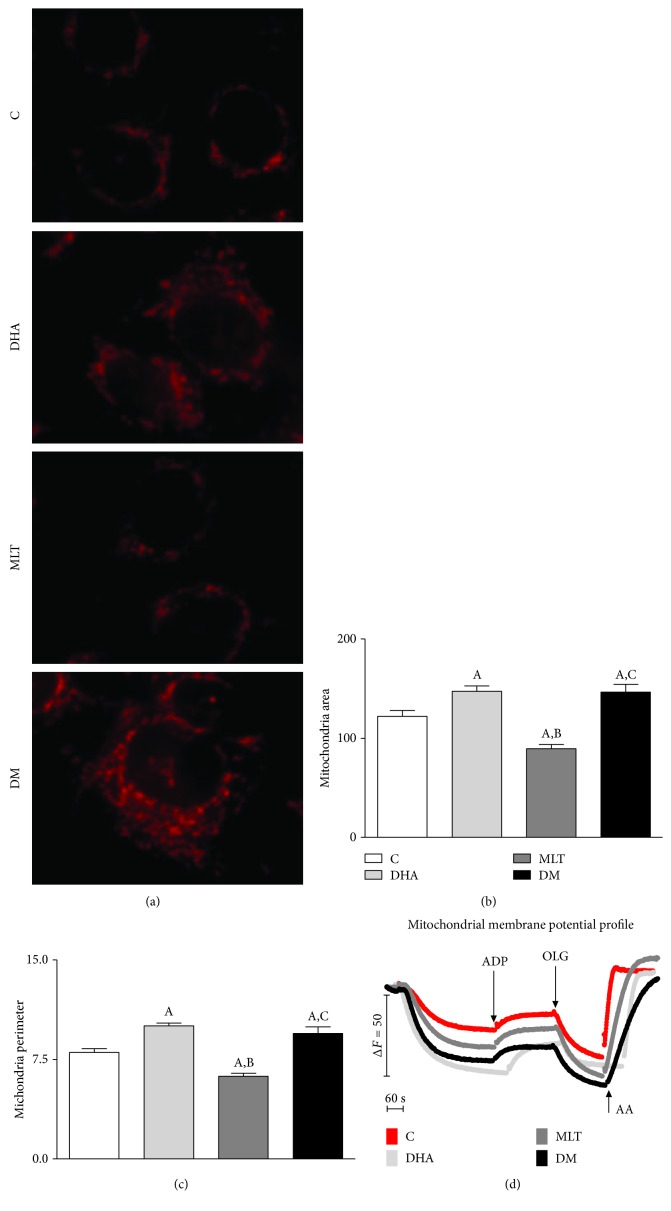
Morphometric analysis of mitochondria. (a) Mitochondria labeling with MitoTracker, (b) area (*μ*m^2^), (c) perimeter (*μ*m), and (d) mitochondrial membrane potential assessed with Safranin O. Images captured with 40x objective with an inverted fluorescence microscope for morphological analysis. Legend: C: control (vehicle incubation); DHA: incubation with 100 *μ*M of DHA for 48 h; MLT: incubation with 1 *μ*M of melatonin for 48 h; DM: coincubation with 100 *μ*M of DHA and 1 *μ*M of melatonin for 48 h; ADP: adenosine diphosphate; OLG: oligomycin; AA: antimycin A. At least 100 cells from consecutive passages were considered for statistical analysis. Values show the mean and SEM. Statistical analysis: (A) different from C; (B) different from DHA; (C) different from MLT. *p* < 0.05 was considered statistically different.

None of the incubations altered basal OCR in PNT1A cells (S2—Figure 4(a)). Respiratory control (Figure 4(b)) was increased by MLT alone or coincubated whereas DHA did not promote any change on OCR when compared to control (control: 3.13 ± 0.08, MLT: 4.89 ± 0.23, and DM: 3.71 ± 0.13). Mitochondrial bioenergetic reserve capacity (MBRC) of PNT1A cells (Figure 4(d)) was unaffected by MLT alone, decreased 2-fold after incubation only with DHA, and raised roughly by 130% in DM compared to control (control: 2.90 ± 0.27, DHA: 1.59 ± 0.11, and DM: 3.77 ± 0.07 nmol O_2_/10^6^ cells/min). MLT alone decreased both mitochondrial area and perimeter, and DHA alone increased as well as coincubation when compared to control (area—control: 122.1 ± 5.79, DHA: 147.1 ± 5.33, MLT: 89.55 ± 4.05, and DM: 146.3 ± 8.04 *μ*m^2^/perimeter—control: 8.02 ± 0.28, DHA: 10.03 ± 0.21, MLT: 6.23 ± 0.23, and DM: 9.47 ± 0.48 *μ*m), as shown in Figures 5(a)–5(c).

### 3.7. MLT and DHA Modulated the Survivor and Cell Proliferation-Related Pathways Regardless of MTR1 or MTR2 Sensitization

**Figure 6:**
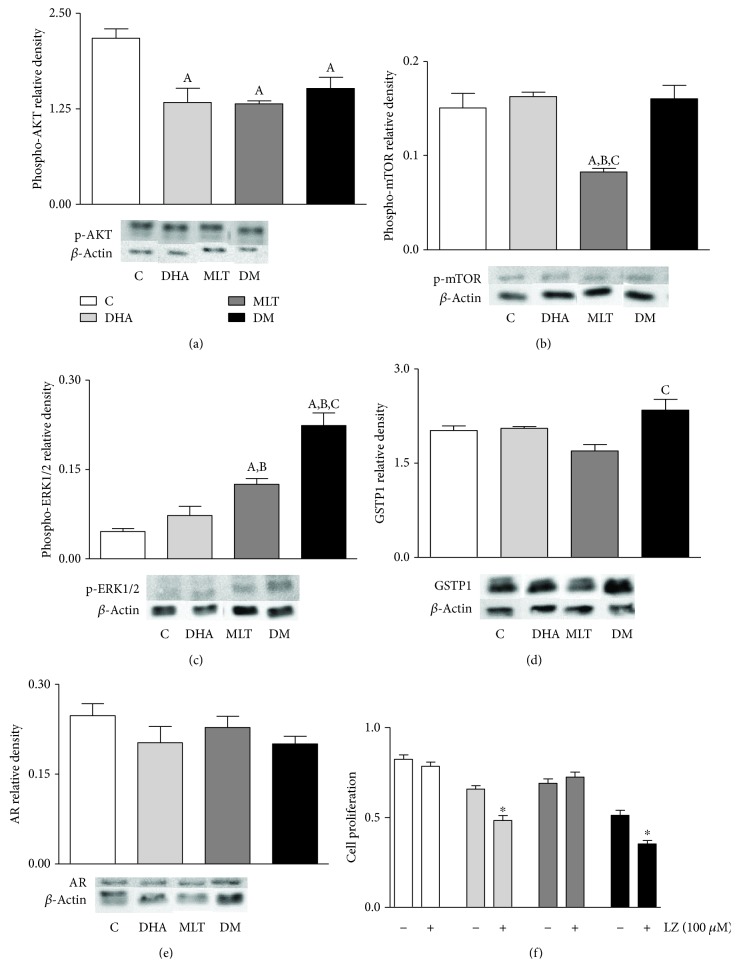
Signaling pathways. (a) AKT, (b) mTOR, and (c) ERK1/2 activation. (d) GSTP1 and (e) AR expression. Legend of panels (a)–(e): C: control (vehicle incubation); DHA: incubation with 100 *μ*M of DHA for 48 h; MLT: incubation with 1 *μ*M of melatonin for 48 h; DM: coincubation with 100 *μ*M of DHA and 1 *μ*M of melatonin for 48 h. Values show the mean of relative density and SEM from three different samples normalized to *β*-actin. For each blotting, the samples run in the same electrophoresis, and except for AR, the bands were cropped, for group standard purposes. Statistical analysis of panels (a)–(e): (A) different from C; (B) different from DHA; (C) different from MLT. (f) Cell proliferation rate after incubation with luzindole. Values show the mean of absorbance and SEM. Legend of panel (f): LZ: luzindole. Statistical analysis of panel (f): ^∗^ different from incubation without luzindole. *p* < 0.05 was considered statistically different. Three independent experiments were performed in triplicate (*n* = 9) for statistical analysis.

Luzindole did not change cell proliferation (Figure 6(f)) in control or MLT, but was reduced when incubated with DHA (control: 0.65 ± 0.02; DHA: 0.48 ± 0.02 abs) and DM (control: 0.51 ± 0.03; DM: 0.35 ± 0.02 abs) compared to incubations without the antagonist.

DHA and MLT alone suppressed AKT phosphorylation (Figure 6(a)) when compared to control, as well as coincubation, which did not show a synergistic effect (control: 2.17 ± 0.12, DHA: 1.33 ± 0.18, MLT: 1.31 ± 0.03, and DM: 1.51 ± 0.15). MLT alone also inhibited mTOR activation (Figure 6(b)) compared to control, whereas DHA alone or in coincubation did not change (control: 0.150 ± 0.015; MLT: 0.082 ± 0.003). On the other hand, MLT alone increased ERK1/2 phosphorylation (Figure 6(c)) compared to control and coincubation with DHA amplified this effect (control: 0.045 ± 0.005, MLT: 0.125 ± 0.009, and DM: 0.223 ± 0.021). GSTP1 expression (Figure 6(d)) did not change in isolated incubations, but together, MLT and DHA increased 20% compared to MLT alone (MLT: 1.69 ± 0.10; DM: 2.34 ± 0.17).

### 3.8. MLT May Influence the Androgen Pathway

**Figure 7:**
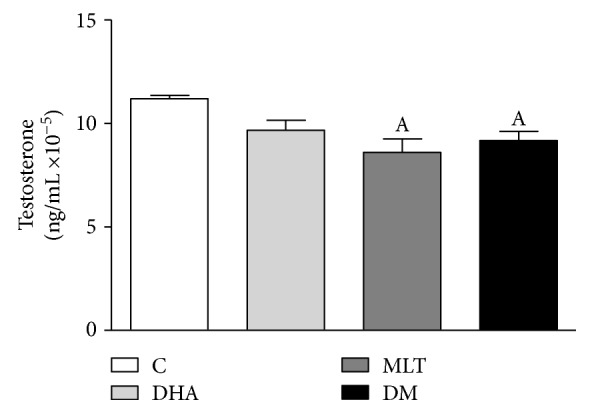
Testosterone level in the medium. After incubations, the medium was removed and testosterone level assessed by ELISA assay. Values show mean of ng of testosterone/mL ×10^−5^ normalized by protein amount from cell lysate and SEM. Legend: C: control (vehicle incubation); DHA: incubation with 100 *μ*M of DHA for 48 h; MLT: incubation with 1 *μ*M of melatonin for 48 h; DM: coincubation with 100 *μ*M of DHA and 1 *μ*M of melatonin for 48 h. Statistical analysis: (A) different from C. *p* < 0.05 was considered statistically different. Three independent experiments were performed in triplicate for statistical analysis.

Testosterone levels in culture medium (Figure 7) decreased by 25% after MLT incubation compared to control and 20% when coincubated with DHA, whereas no alteration was observed for DHA alone (control: 11.20 ± 0.15, MLT: 8.60 ± 0.64, DM: 9.18 ± 0.43 ng/mL × 10^−5^). AR expression did not change among any incubation (Figure 6(e)).

## 4. Discussion

Considering that the available strategies against PCa are still highly inefficient and may lead to the recurrence of more aggressive phenotypes [22, 23], we investigated, *in vitro*, the potential of DHA and MLT to decrease cell survival at early stages of proliferative disorders. PNT1A cells were chosen due to their normal secretory phenotype but also nonmalignant alterations, allowing study growth and survival of high proliferative prostate cells which are sensitive to androgen, as described in early stages of prostate cancer progression and BHP [33]. Furthermore, this cell line retains AR expression, whose activity is closely related to cell metabolism, and the loss thereof affects mitochondria yields and dynamics among PCa progression. Due to their antiproliferative properties, DHA and MLT have been investigated as PCa suppressors in experimental studies [11, 18], but their combined action in benign prostate cells has not yet been evaluated. In this study, we observed that ROS generation and mitochondrial function play a pivotal role in determination of PNT1A cell proliferation. On the one hand, DHA had an anticlonogenic effect at the highest concentration tested (100 *μ*M) after 48 h of incubation, mainly due to the increase in ROS production, probably related to mitochondrial function impairment and AKT inactivation. On the other hand, MLT, at the pharmacologic range, improved oxidative phosphorylation and decreased ROS production, but reduced cell proliferation due to inhibition of survival pathways. Moreover, MLT improved the DHA antiproliferative effect in PNT1A cells.

### 4.1. Benign Epithelial Prostatic Cells Respond Differently to DHA than Tumor Cells

**Figure 8:**
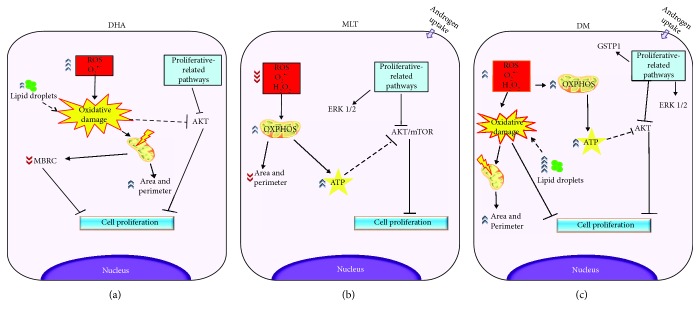
Proposed mechanism. (a) DHA increased ROS production, mainly O_2_^●−^, leading to oxidative damage that may trigger mitochondrial fusion as tentative to restoration of organelle function. Also, such fatty acid increased lipid accumulation which may increase ROS. In this context, we hypothesize that DHA raised oxidative stress, therefore impairing mitochondrial function, which then stimulated organelle fusion and led to area and perimeter increase as well as the recovery of OXPHOS, but not MBRC. In addition, DHA also inhibited AKT activation which may be related to increase in oxidative damage and decrease in cell proliferation. (b) MLT decreased ROS production, both O_2_^●−^ and H_2_O_2_, and improved OXPHOS which may be associated to AKT/mTOR inactivation. (c) DHA increased oxidative damage which was not neutralized by MLT, but together (DM) amplified the antiproliferative effect probably due to AKT and ERK1/2 regulation. MLT, alone or coincubated, stimulated ERK1/2 activation as well as androgen uptake, but the mechanism is not clear. Also, MLT coincubated with DHA-stimulated GSTP1 expression probably due to ROS increase. Legend: DHA: docosahexaenoic acid; MLT: melatonin; DM: coincubation; O_2_^●−^: superoxide anion production; H_2_O_2_: hydrogen peroxide; ATP: adenosine triphosphate; OXPHOS: oxidative phosphorylation; MBRC: mitochondrial bioenergetics reserve capacity; ROS: reactive oxygen species. Dashed lines, mechanism proposed based on the literature; solid lines, effects and correlations based on observed results.

DHA has been described to have antimitotic and proapoptotic effects on malignant prostate cell lines using different concentrations [18, 34, 35]. However, in this study DHA had a hormetic behavior on nontumor PNT1A cells, since an inhibitory effect on proliferation was detected at a higher concentration (100 *μ*M) for longer exposures (48 and 72 h), whereas a proliferative stimulus was triggered at lower concentrations (20 and 50 *μ*M). Despite not being the aim of our study, it is worth mentioning that the results from our laboratory (not shown) with DHA 50 *μ*M for 48 h suggest that such an increase in cell proliferation may be related to deregulation of peroxisome proliferator-activated receptor gamma (PPAR*γ*), which is still under investigation. Regarding DHA at 100 *μ*M for 48 h, it is possible to suggest two key factors in cytotoxic effect: the increase in ROS production and the inactivation of AKT (Figure 8(a)). Interestingly, previous studies demonstrated that 50 *μ*M of DHA led to death of PC3 and DU145 cells [18] whereas in our study this concentration stimulated PNT1A to proliferate and cytotoxic effects were observed solely at 100 *μ*M. In PCa, oxidative stress is increased and it is possible that incubation with DHA enhanced cellular damage, activating cell death even at lower concentrations. Furthermore, this susceptibility may also rely on metabolic changes among malignant transformations known as the Warburg effect [36]. Regarding PCa, these changes may be related to several mechanisms [37], as increased *β*-oxidation [38], higher amounts of mitochondria and lower complex I activity [4], mutations in ETC complexes [5], and alterations in mitochondria dynamics [39] which may be related to different responses between benign and prostate tumor cells.

### 4.2. DHA Raised ROS Production and Mitochondrial Rearrangement

To the best of our knowledge, this is the first evidence that DHA increases ROS generation and decreases cell proliferation in PNT1A benign prostate cells. Such effects were probably associated to higher O_2_^●−^ production, which is related to the increase in mitochondrial membrane potential (Figure 5(d)) [40]. It is currently known that higher oxidative stress levels commonly exhibit impairment of mitochondrial activity [41]. In response to oxidative damage or metabolic changes, mitochondrial fusion may be triggered as tentative to restoration of organelle function [42]. In this context, we hypothesize that DHA raised oxidative stress, therefore impairing mitochondrial function, which then stimulated organelle fusion and led to area and perimeter increase as well as the recovery of oxidative phosphorylation (OXPHOS). Mitochondrial morphology alterations described here are in line with previous reports, since DHA upregulated the protein related to mitochondrial fusion MFN2 [43]. It is worth mentioning that despite that OXPHOS was unchanged after 48 h of incubation, mitochondrial integrity probably remained damaged because DHA strongly impaired MBRC, an index of cell potential to respond upon stress conditions, which is decreased under oxidative stress [44]. In this context, *in vitro* studies from our research group found that half of DHA concentrations used here remarkably decreased the viability of the testis cell population in fetal mice and, when combined to 2-monoethylhexyl phthalate (MEHP), strongly impaired the survival of male germ cells (gonocytes). Therefore, the data highlights the ability of DHA to induce cell sensitization to other cytotoxic compounds (data not published).

### 4.3. Decrease in ROS Production by MLT Alone Is Associated to Lower Cell Proliferation Rates

Because oxidative stress apparently is a key factor for cell survival [18], we evaluated the antioxidant potential of MLT. MLT is an expected antioxidant agent which can scavenge free radicals directly or by its metabolites [45], upregulate the expression and activity of antioxidant enzymes, such as superoxide dismutase, glutathione peroxidase, and catalase [46], or modulate mitochondrial activity [6, 47]. In the present study, our aim was to validate on PNT1A cells the MLT antioxidant action and not the mechanisms per se. Indeed, MLT was able to decrease H_2_O_2_ and O_2_^●−^ production, combined or not to DHA. Our data are in line with previous literature, since it was reported that MLT, at the same concentration, decreased ROS in isolated mitochondria from the liver [10]. Such an effect on oxidative stress attenuation may be associated to the attenuation of proton leak in mitochondria [48] or even decrease in mitochondrial membrane potential, a well-known contributor to O_2_^●−^ production *in vitro* and *in vivo* [40]. Levels of ROS production are closely related to OXPHOS [6], and decrease in H_2_O_2_ and O_2_^●−^ described may be related to improvement of the respiratory control ratio. It is worth mentioning that complex II-supported respiration was chosen based on the fact that such complex is invariable among cancer cells [49] and it is an important site for ROS generation, while it also contributes to ROS either directly or indirectly via reverse electron transfer [50]. To our knowledge, this is the first report to show antioxidant properties of MLT in PNT1A cells associated to improvement of the mitochondrial physiology. Because the increase in ROS generation had been related to expansion of mitochondrial area and perimeter, we investigated if the decreased production of reactive species associated to OXPHOS improvement also affected organelle morphology. Thus, it was possible to observe that mitochondrial function enhancement was followed by decrease in organelle area and perimeter. The findings herein suggest a quality control system that removes fractions or the entirety of mitochondria with higher ROS generation which may be harmful to organelle homeostasis. On the other hand, we cannot discard that MLT effects on mitochondria physiology may be a consequence of first changes in organelle dynamics, as well as contribution of antioxidant system enzyme modulation. Therefore, the present study highlights new possibilities of MLT's role as a regulator of mitochondrial function in prostatic epithelial cells.

The results observed here regarding proliferation of PNT1A cells were different from the previous results [51], since it was reported that MLT at 500 *μ*M or 10 mM did not affect cell proliferation. Such differences may be due to conditions of incubation but also to higher cell density seeded (10^4^ cells/well) than what Gobbo and colleagues [51] performed (3 × 10^3^ cells/well), which may be more suitable to clarify the differences between proliferation rates among incubations. In the present study, OXPHOS improvement and decrease in ROS production by MLT was probably associated to AKT inactivation and decrease in cell proliferation. Our data may suggest an association with ATP, since its increase may be associated to cell death [52]. MLT's property of modulating ATP levels was reported at different concentrations and systems due to several mechanisms, such as a raise in ADP/O ratio and upregulation of *β* subunit of ATP synthase [7–10]. Despite not being assessed, it is suggested that OXPHOS improvement may be related to increase in ATP levels in the cells. This hypothesis is in line with previous literature, since MLT in the liver and brain tissue, even at lower concentrations (100 nM), was able to improve OXPHOS through stimulation of complexes I and IV and protection of these enzymes against oxidative damage, which was closely related to increase in ATP production [7]. In this context, the increase in ATP levels may suppress the AKT pathway [53] and decrease survival, as observed here and reported in breast cancer cells after administration of extracellular ATP to the medium [54]. Therefore, this hormone decreased cell proliferation probably due to the decrease in ROS production that affected pathways of mitosis stimulation and cell survivor. PCa progression is related to the rise in oxidative stress and, together with our data, point to mitochondrial bioenergetics' role on cell arrest and suggest that MLT is effective as antiproliferative agent acting in this pathway (Figure 8(b)).

### 4.4. MLT Antioxidant Ability Apparently Is More Efficient at the Mitochondrial Compartment

Higher lipid accumulation probably was related to increased amounts of DHA. Such omega-3 is metabolized primarily at the peroxisome fraction, a well-known site of H_2_O_2_ generation, due to long-chain fatty acid structure [19]. Regarding coincubation, H_2_O_2_ production was increased and MLT was not able to decrease it. Therefore, the antioxidant effects of MLT in the presence of DHA were mainly at the mitochondrial compartment since, despite not recovering to control levels, such hormone decreased O_2_^●−^ levels possibly related to OXPHOS improvement in DM. In addition, such decrease may attenuate mitochondrial oxidative damage, which can be related to the enhancement of MBRC observed in coincubation.

### 4.5. DHA and MLT Downregulate Cell Proliferation and Survival Pathways

In PC3 and DU145 lines, DHA led to AKT/mTOR suppression [18], whereas in PNT1A only the activation of AKT decreased. This evidence may suggest a different action of such omega-3 when distinct stages of proliferative disorders are considered. Concerning MLT alone, it suppressed the AKT/mTOR pathway, which is closely related to decrease in cell proliferation. However, when coincubated to DHA, MLT enhances the decrease in proliferation by activating ERK1/2. Despite being generally associated to proliferation stimulus, activation of ERK1/2 may also lead to cell death mainly under stress conditions [55]. In PC3 cells, the increase in oxidative stress reduced AKT activation, followed by increased phosphorylation of ERK and cell proliferation arrest [56], similar to what was observed in PNT1A in our study after coincubation. Despite the fact that DHA alone did not stimulate ERK1/2 activation, even under high ROS production, in our *in vitro* model, this evidence highlights that MLT could play a key role in the modulation of such pathway. In addition, we also suggest that MLT combined to increased oxidative stress, the ERK1/2 pathway is strongly activated and therefore controls cell survival, as reported previously [57]. It is worth mentioning that higher levels of ERK activation may also be related to GSTP1 upregulation [58].

Because androgen regulation is important for the survival of prostate cells and PCa development, we analyzed if incubation effects were related to the alteration in this pathway. In the present incubations, DHA did not change testosterone levels in medium or AR expression, which supports the hypothesis that the antimitotic property of this omega-3 in PNT1A cells was mainly due to alterations on oxidative status. AR expression was also unchanged after MLT exposure, regardless of DHA incubation. However, MLT influenced the androgen levels in the medium, but due to methodologic limitations, it is not clear if the release of testosterone or its uptake was affected by MLT. Furthermore, Estrada and colleagues [59] demonstrated that in the presence of androgen, ERK activation was increased and followed by cell proliferation decrease. Therefore, our study suggests that MLT may improve antiproliferative effects due to an association between mitochondrial bioenergetics, androgen pathway, and ERK pathway, even in nontumor cells (Figure 8(c)), although additional studies are needed to better understand this issue.

Finally, we proved that PNT1A proliferation, when incubated with MLT, was independent of MTR1 and MTR2 activation, both expressed in the prostate gland [60]. This demonstrates that MLT uptake by PNT1A cells, already reported previously [26], activated pathways regardless of membrane receptors and had a key role in mitochondrial activity modulation and oxidative stress control.

## 5. Conclusion

Our data indicates that PNT1A cells are less sensitive to DHA than advanced cancer cell lines; however, at 100 *μ*M this omega-3 reduced the proliferation rate due to the increase in ROS production at cytotoxic levels and possibly associated to the inhibition of AKT activation. It is important to emphasize DHA hormetic behavior in PNT1A cells, because at lower concentrations it stimulated cell proliferation. This finding highlights the need for further studies, concerning dietary supplementation with this PUFA, mainly in men at risk of PCa development. Furthermore, MLT at 1 *μ*M increased OXPHOS, decreased ROS production, and exerted an antimitogenic effect via AKT inactivation and ERK1/2 activation regardless of binding in MTR1 or MTR2. Moreover, MLT improved *in vitro* DHA antiproliferative effect, which may open new possibilities of chemopreventive strategies, mainly at early stages of prostate proliferative disorders.

## Data Availability

The data used to support the findings of this study are available from the corresponding author upon request.
